# Ectopic Crossed Testis: A Rare Anomaly of Testicular Migration

**DOI:** 10.7759/cureus.39086

**Published:** 2023-05-16

**Authors:** Bhagyasri Nunna, Pratap Parihar, Harshith Gowda, Vadlamudi Nagendra, Nidhi G Reddy

**Affiliations:** 1 Department Radiodiagnosis, Jawaharlal Nehru Medical College, Datta Meghe Institute of Higher Education & Research, Wardha, IND

**Keywords:** ipsilateral inguinal hernia, magnetic resonance imaging, ultrasonography, cryptorchidism, ectopic crossed testis

## Abstract

Ectopic crossed testis is a rare condition in which both testes descend through the same inguinal canal. The most common presentation is an ipsilateral inguinal hernia with contralateral cryptorchidism. This is a case report of a six-year-old male child who had an empty right scrotal sac. Diagnostic laparoscopy is useful for both diagnosis and management. Management is determined by the anatomy of the vas, vessels, and testis discovered during surgical exploration. Transseptal contralateral orchidopexy results in good tension-free testicular fixation in the scrotum.

## Introduction

Testes may be ectopic in the abdomen and inguinal area and can also descend into the contralateral scrotal sac [1]. Crossed testicular ectopia is a rare condition characterized by the migration of both testes down a single inguinal canal. The anatomy of the testis, blood vessels, and vasculature found during the surgical investigation is used to guide treatment [2]. In situations when crossed ectopic testis can be mistaken for undescended testis, imaging tests are more beneficial than laparoscopic inspection because a lack of awareness results in a delay in diagnosis and unintended detection during surgery [3].

## Case presentation

A six-year-old male child presented to the hospital complaining of an empty right scrotum since birth and swelling on the left side of the scrotum. According to the mother, she noticed the empty right scrotum shortly after the child's birth and consulted a private doctor, who advised observation only then. When the child reached the age of six years, he directly presented to the doctor with a complaint of an empty right scrotum and swelling on the left side of the scrotum.

**Figure 1:**
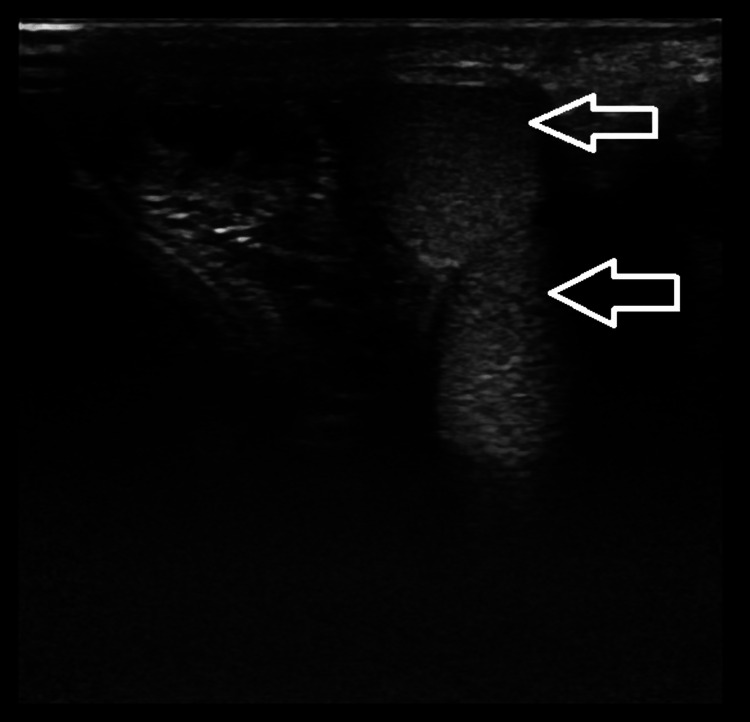
Gray-scale ultrasound image showing one testis in the left scrotal sac with another testis (white arrows) at the root of the left scrotum.

**Figure 2:**
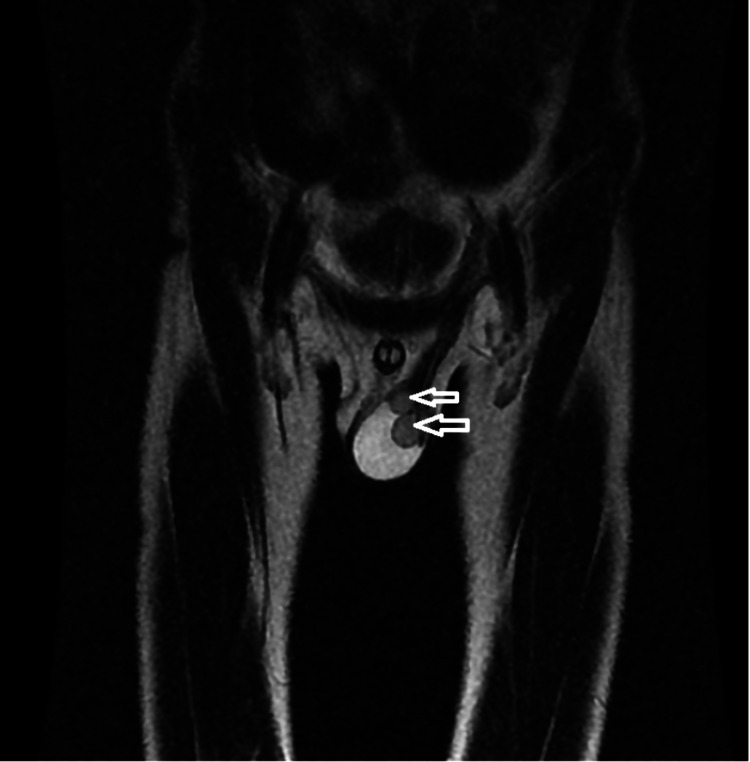
T2WI MRI image coronal section showing moderate left-sided hydrocele with left testis and another testis at the root of the scrotum on the left side (white arrows). T2WI: T2-weighted image.

Upon examination, the doctor found that the right scrotum was empty, and the left testis was palpable. There was also swelling on the left side of the scrotum. To confirm the diagnosis, an ultrasound (USG) was performed in the inguinoscrotal region, which revealed two testes on the left side, an empty right scrotum suggestive of an undescended right testis, and a left-sided crossed ectopic testis, as shown in Figure 1. Subsequently, an MRI was performed, which revealed an empty right scrotum, moderate left hydrocele, and another testis located at the root of the left scrotum, as shown in Figure 2. This testis was most likely an ectopic undescended testis, and the possibility of ectopic crossed testes was also given. Transseptal orchiopexy was performed, resulting in the fixation of the testis, located at the root of the left scrotum, within the right scrotum.

## Discussion

A rare condition known as transverse testicular ectopia (TTE) is marked by the migration of both testes to the same hemi-scrotum. Less than 65% of instances of TTE are detected prior to surgery, which is a low incidence. The majority of instances (65%) are often found when an inguinal hernia is repaired [4]. Based on the accompanying anomalies, there are three different forms of crossed testicular ectopia: inguinal hernias are the only cause of type 1, which makes up 40%-50% of cases; type 2 or persistent or rudimentary Mullerian duct structures; and type 3 or other genitourinary abnormalities without Mullerian remnants [5].

A recommended surgical treatment option is orchiopexy to preserve fertility following the identification of TTE. TTE and related abnormalities can be diagnosed and treated well using laparoscopy [6]. Transseptal orchiopexy and extraperitoneal transposition orchiopexy are the two most often performed surgical procedures to repair testes in the scrotum. The testis is transported to the opposing hemi-scrotum by extraperitoneal transposition orchiopexy after passing close to the penis root [7].

## Conclusions

In cases of undescended testes, the condition of the crossed ectopic testis is a rare phenomenon that requires diagnosis prior to surgery. It is recommended to undertake magnetic resonance imaging (MRI) and/or ultrasonography (USG) in cases when there is a suspicion of such a problem before performing a diagnostic laparoscopy. Both the diagnosis and treatment of the illness are aided by this strategy.
